# Early radiobiological effects of flattening filter (FF) and flattening filter-free (FFF) beams and the radioprotective role of melatonin in rat brain: a preclinical study

**DOI:** 10.1093/jrr/rraf078

**Published:** 2025-12-23

**Authors:** Şule Baz Baz Çifci, Serhat Aras, Fatih Hacımustafaoğlu, Esra Erdem, Navid Kheradmand, Mustafa Çağlar

**Affiliations:** Department of Radiation Oncology, Faculty of Medicine, Gaziantep University, Üniversite Bulvarı 27310, Şahinbey, Gaziantep, Türkiye; Department of Health Physics, Graduate School of Health Sciences, Istanbul Medipol University, Kavacik District, Ekinciler Street No: 19, 34810 Beykoz, Istanbul, Türkiye; Department of Radiation Oncology, Haydarpasa Numune Training and Research Hospital, Tibbiye Street No: 40, 34668 Uskudar, Istanbul, Türkiye; Medical Imaging Techniques Program, Department of Medical Services and Techniques, Hamidiye Vocational School of Health Services, University of Health Sciences, Selimiye District, Tibbiye Street No: 38, 34668 Uskudar, Istanbul, Türkiye; Medical Laboratory Techniques Program, Hamidiye Vocational School of Health Services, University of Health Sciences, Selimiye District, Tibbiye Street No: 38, 34668 Uskudar, Istanbul, Türkiye; Department of Histology and Embryology, Hamidiye Faculty of Medicine, University of Health Sciences, Selimiye District, Tibbiye Street No: 38, 34668 Uskudar, Istanbul, Türkiye; Radiotherapy Program, Vocational School of Health Services, Istanbul Medipol University, Ekinciler Street No: 19, Kavacik District, 34810 Beykoz, Istanbul, Türkiye; Department of Health Physics, Graduate School of Health Sciences, Istanbul Medipol University, Kavacik District, Ekinciler Street No: 19, 34810 Beykoz, Istanbul, Türkiye

**Keywords:** radiotherapy, FFF, brain, histopathology, biochemical, melatonin

## Abstract

The study aimed to investigate early radiobiological effects of Flattening Filter (FF) and Fattening Filter-Free (FFF) beams on brain tissue in experimental rat models and to evaluate the potential radioprotective role of melatonin (MEL) using histopathological and biochemical parameters. Forty female Wistar albino rats were randomly assigned to five groups: control (G1), FF (G2), FF + MEL (G3), FFF (G4) and FFF + MEL (G5). A single 16 Gy dose was delivered to the head and neck region in G2 and G4. MEL (50 mg/kg) was administered intraperitoneally 15 minutes before irradiation in G3 and G5. Forty-eight hours post-irradiation, serum samples were analyzed for M30, M65, TAS, TOS and OSI. Brain and cerebellar tissues were histologically examined for neuronal degeneration, vascular dilatation, congestion, axonopathy, inflammation, dysplasia and necrosis. While M30, M65, TOS and OSI levels increased in G2 and G4 radiotherapy groups, TAS levels decreased for biochemical analyses (*P* < 0.05), reflecting impaired antioxidant capacity. However, these alterations were significantly reduced in the MEL-treated groups (G3 and G5) (*P* < 0.05). Histopathologically, neuronal degeneration, vascular dilatation and congestion were observed in G2 and G4 groups. MEL administration significantly alleviated these findings. No statistically significant differences were found between the FF and FFF groups regarding biochemical or histopathological outcomes (*P* > 0.05). While FF and FFF beams caused similar levels of oxidative stress and histopathological damage in the brain tissue, MEL treatment significantly reduced these damages. MEL emerges as a promising radioprotective agent against early radiation-induced brain injury.

## INTRODUCTION

Cancer remains a significant global health challenge, and radiotherapy is an important part of its management, with approximately two-thirds of all cancer patients undergoing radiation treatment [1, 2]. Technological advancements in radiotherapy devices have played an important role in improving treatment efficacy and precision [3]. Among these innovations, the clinical implementation of flattening filter-free (FFF) technology—achieved by removing the flattening filter (FF) from the linear accelerator head—has introduced several dosimetric and clinical advantages [4, 5]. One of the key advantages of FFF beams is that they have smaller penumbra compared to conventional FF beams, which allows for sharper dose gradients and improved target compatibility. Furthermore, FFF technology has been suggested to contribute to a reduced risk of radiation-induced secondary malignancy, primarily due to a lower scattering dose to surrounding healthy tissues; however, it should be noted that this conclusion is largely based on dosimetric modeling studies rather than long-term clinical outcome data [6, 7]. In particular, FFF-enabled linacs can deliver dose rates up to three to four times higher than conventional FF-based systems. This increase in dose rate leads to significantly shorter irradiation times, improving patient comfort and reducing the likelihood of intrafractional motion [8–10].

Exposure of biological tissues to ionizing radiation leads to the formation of reactive oxygen species (ROS), which play a central role in radiation-induced cellular damage. High levels of ROS contribute to oxidative stress by producing free radicals that target critical cellular components such as DNA, proteins and lipids [11, 12]. Antioxidants combat radiotherapy-induced oxidation and mitigate oxidative stress resulting from free radicals generated by radiation treatment. Melatonin (MEL) was first identified in 1958 as a pineal gland hormone and has since been recognized as a powerful antioxidant and free radical scavenger with therapeutic effects against radiation-induced damage [13, 14]. Beyond its role as a systemic antioxidant, MEL has the unique ability to cross the blood–brain barrier, making it a particularly relevant candidate for protecting central nervous system tissues against radiation-induced oxidative stress. [13, 15] Several studies have confirmed its protective effects by demonstrating reductions in oxidative stress, mitochondrial dysfunction and apoptosis in irradiated tissues [16, 17]. Several studies confirmed the protective effects of MEL by demonstrating its ability to reduce oxidative stress and inhibit apoptosis in irradiated tissues [16, 17]. Radiotherapy to the brain is associated with several radiation-induced adverse effects, such as cerebral edema and necrosis, which reflect damage to normal brain tissue and constitute dose-limiting factors in clinical practice. Although MEL has been investigated as a potential radioprotector in central nervous system models, previous studies have reported variable results, with some demonstrating significant neuroprotection and others showing limited benefit. These discrepancies highlight the need for further experimental evidence. These effects reflect damage to normal brain tissue and are important dose-limiting factors in clinical practice [18]. Radiation-induced damage in organs at risk is usually assessed through a combination of histopathological and biochemical markers, which primarily reflect oxidative stress caused by high levels of free radicals. In parallel, biochemical analyses were performed on serum samples to measure markers associated with cell death and oxidative stress. These included M30 (a marker of apoptosis), M65 (a marker of necrosis), total oxidant status (TOS) and total antioxidant status (TAS).

The primary aim of this study was to investigate possible radiobiological differences in the biochemical and histopathological responses of healthy cerebellum and cerebral cortex tissues in Wistar albino rats following irradiation with FF and FFF beams at varying dose rates, in the absence of tumor burden. This direct comparison of FF versus FFF beams on early brain tissue response represents, to our knowledge, one of the first experimental studies in this area. A secondary aim was to evaluate the radioprotective efficacy of MEL by examining its modulatory effects on radiation-induced tissue damage, as assessed by both histopathological changes and biochemical markers.

## MATERIALS AND METHODS

### Experimental animals, experimental groups and MEL administration

Following approval by the Istanbul Medipol University Animal Experiments Local Ethics Committee (Approval No. E-38828770-772.02-7477), a total of 40 healthy female Wistar albino rats (6–8 weeks old, weighing 220 ± 20 g) were included in the study. All experimental procedures were conducted in accordance with institutional and national guidelines for the care and use of laboratory animals. Animals were housed under controlled environmental conditions, including 60% ± 10% relative humidity, a constant temperature of 20 ± 1°C, and a 12 h light/12 h dark cycle.

Rats were randomly assigned into five groups with eight animals in each group (Table 1). A non-irradiated MEL-treated control group was not included, as the primary aim of this study was to investigate irradiation-related differences. MEL (50 mg/kg; Melatonin Crystalline, Sigma-Aldrich Corporation, St. Louis, MO, USA) was dissolved in 1% ethanol–physiological saline solution and administered intraperitoneally 15 min before irradiation, followed by a single dose of radiotherapy using both G2 and G4 [19, 20].

**Table 1:** Experimental animal groups and procedures for radiotherapy and melatonin administration

Groups	Radiotherapy and melatonin administration procedures
G1	No irradiation or melatonin administered
G2	Single 16 Gy irradiation at 600 MU/min using a 6 MV X-ray with flattening filter (FF)
G3	IP injection of melatonin (50 mg/kg, 15 min before RT) + single 16 Gy irradiation at 600 MU/min (FF)
G4	Single 16 Gy irradiation at 2400 MU/min using a 6 MV X-ray with flattening filter-free (FFF) beam
G5	IP injection of melatonin (50 mg/kg, 15 min before RT) + single 16 Gy irradiation at 2400 MU/min (FFF)

### Radiotherapy procedure

In this study, all rats were anaesthetized by intraperitoneal (IP) injection of 80 mg/kg ketamine and 5 mg/kg xylazine to ensure immobility during irradiation. Each rat was positioned and immobilizated on the radiotherapy setup in the supine position. Radiotherapy was administered at a source-to-surface distance (SSD) of 100 cm with the gantry angle fixed at 0°. A 5 × 5 cm^2^ irradiation field was designed to cover the head and neck region of the rats, ensuring that the entire region remained within the treatment field.

Before irradiation, machine output factor was verified to deliver 1 cGy per monitor unit (1 MU = 1 cGy) under reference conditions. Irradiation was performed with a Varian TrueBeam linear accelerator using 6 MV X-rays and 6 MV FFF X-rays. Each rat received a single 16 Gy dose to the head and neck region. Two different dose rates were applied: 600 MU/min for the FF beam and 2400 MU/min for the FFF beam. Identical field arrangements were used for both groups to minimize beam profile differences.

To correct for surface irregularities and improve dose homogeneity, a 10 mm tissue-equivalent bolus was placed over the irradiated neck region. Dose distributions between FF and FFF plans were verified to be within ±3% across the irradiated volume, ensuring that the only variable between FF and FFF groups was the dose rate.

### Euthanasia and tissue collection

Forty-eight hours following the completion of radiotherapy, all rats were euthanized under deep anesthesia, induced by IP administration of 80 mg/kg ketamine and 5 mg/kg xylazine. Cardiac blood samples were collected immediately after euthanasia and the obtained samples were centrifuged to isolate serum. Serum samples were aliquoted into Eppendorf tubes and stored at −80°C for subsequent biochemical analyses. After blood collection, brain and cerebellum tissues were carefully dissected. The collection of tissues was fixed in 10% formaldehyde solution.

### Histopathological analysis

Histological examinations were performed in the Department of Histology and Embryology, Hamidiye Medical Faculty, University of Health Sciences (SBU). The cerebral cortex and cerebellum tissues were fixed in 10% formaldehyde at a temperature of 4°C for a duration of 48 hours. After fixation, the samples were subjected to routine histological procedures including dehydration, clearing and paraffin embedding. Paraffin embedded tissues were then sectioned for microscopic evaluation. Tissues prepared as paraffin blocks were sectioned with a microtome at a thickness of 4 μm. To remove wrinkles, it was placed in a water bath (40°C). Sections stained with hematoxylin and eosin (H&E) were evaluated by taking photographs under a Zeiss brand light microscope with a digital camera attachment (Axiocam). Morphological evaluation included assessment of neural degeneration, axonopathy, inflammation, dysplasia, vascular dilatation and congestion in the cerebral cortex. In the cerebellum, neural degeneration, vascular dilatation, congestion and neuronal cell necrosis were evaluated. All findings were compared with the control group to determine histopathological changes attributable to irradiation.

Histopathologic analyses were performed by the same randomized blinded examiner for each group. In each section, histologic parameters were scored by a single person using semiquantitative method similar to Aras *et al.* 0: no damage, 1: mild damage, 2: moderate damage and 3: severe damage [21]. This scoring system was applied to all predefined histological findings in both cerebral cortex and cerebellum tissues to allow consistent intergroup comparison of radiation-induced pathological changes. For each histological parameter, median [minimum–maximum] scores per group were calculated and presented in tabular form.

### Biochemical analysis

Blood from all rats was transferred into serum tubes and allowed to clot for 30 minutes. Serum was then separated by centrifugation at 1500 × g for 10 minutes. Sera were transferred into microcentrifuge tubes and stored at −80°C until the day of analysis. TAS, TOS, OSI, M30 and M65 levels were analyzed in the serum samples obtained. In addition, correlations between serum biomarkers (M30, M65) and histopathological scores were analyzed.

**TAS:** TAS was measured on a [BMG LABTECH, Ortenberg, Germany] fully automated chemistry analyzer. In this method, the blue-green colored 2,2′-azinobis-(3-ethylbenzothiazoline-6-sulfonic acid) radical cation (ABTS^.+^) is reduced by the antioxidants in the sample, causing a color loss. The resulting color change is measured as absorbance change at a wavelength of 660 nm. Results are expressed as mmol Trolox eq./L [22].

TOS: TAS was measured on a BMG LABTECH, Ortenberg, Germany fully automated chemistry analyzer. In the test, oxidants oxidize Fe^2+^ ions to Fe^3+^; the ferric ions formed form a complex with xylenol orange. The color change is measured at 560/800 nm and the results are expressed as μmol H₂O₂ eq./L [23].

Oxidative stress index (OSI): OSI, as an indicator of oxidative stress, is calculated as the percentage ratio of TOS level to TAS level. This ratio numerically expresses the oxidative and antioxidative balance. During the calculation, the TAS unit was converted from mmol Trolox Eq./L to μmol Trolox Eq./L and the OSI value was calculated according to the formula below and given in arbitrary units (AU).

$\mathrm{OSI}\ \left(\mathrm{AU}\right)=[\mathrm{TOS}\ \left(\mathrm{\mu} \mathrm{mol}\ \mathrm{H}_{2}\mathrm{O}_{2}\mathrm{Eq}./\mathrm{L}\right)/\mathrm{TAS}\ (\mathrm{\mu} \mathrm{mol}\ \mathrm{Trolox} \mathrm{Eq}./\mathrm{L})]\ast 100$ [24]

M30 and M65: M30, used for apoptosis assessment, and M65, used for determination of total cell death reflecting both apoptosis and necrosis, were analyzed by a double antibody-based sandwich ELISA according to the manufacturer’s protocol (YL Biont, Shanghai, China). [25] Commercial kits with catalog numbers YLA0547RA (M30) and YLA0892RA (M65) were used.

Samples were introduced to the plate coated with antibodies, which functions to attach the targeted antigen to the antibody. Thereafter, a biotinylated antibody was introduced to the target antigen present in the sample. The addition and binding of Streptavidin-HRP to a biotinylated antibody has been achieved. Unbound Streptavidin-HRP was removed during the washing step after incubation. Subsequently, the substrate solution was introduced, following which a color change corresponded to the quantity of antigen present. The process was halted by the addition of an acidic stop solution, and absorbance was quantified at 450 nm. All of the steps were performed based on the manufacturer's instructions.

### Statistical evaluation

All statistical analyses were performed using SPSS Statistics v26 (IBM Corp., Armonk, NY, USA) software. The compatibility of the variables used in the study with normal distribution was evaluated by Shapiro–Wilk test. For normally distributed variables, comparisons between more than two groups were made with one-way analysis of variance; in case of a significant difference, Tukey post-hoc test was applied to determine the differences between the groups. For variables that did not show normal distribution, comparisons between more than two groups were performed with the Kruskal-Wallis test, and in cases where significance was found, pairwise group comparisons were made with the Mann–Whitney U test. Correlation analyses between biochemical markers (M30, M65, TOS, TAS, OSI) and histopathological scores were performed using Spearman’s or Pearson’s correlation coefficients depending on data distribution. Two-sided *P*-values <0.05 were considered statistically significant in all analyses.

## RESULTS

### Biochemical findings

The serum levels of oxidative stress markers (TAS, TOS, OSI) and apoptosis-related biomarkers (M30, M65) for all experimental groups are summarized in Table 2. In the G2 and G4 only radiation treatment groups, significant changes were observed compared to the G1.

**Table 2:** Serum levels of TAS, TOS, OSI, M30 and M65 in experimental groups

Parameter	G1	G2	G3	G4	G5
TAS (mmol Trolox Eq./L)	1.37 ± 0.20^a^^,^^c^	1.10 ± 0.05^a^^,^^b^	1.38 ± 0.13^b^	1.05 ± 0.19^c^^,^^d^	1.30 ± 0.12^d^
TOS (μmol H₂O₂ Eq./L)	12.05 ± 3.91^a^^,^^c^	16.52 ± 2.93^a^^,^^b^	11.87 ± 0.89^b^	16.78 ± 2.18^c^^,^^d^	12.25 ± 1.33^d^
OSI (AU)	0.90 ± 0.33^a^^,^^c^	1.51 ± 0.31^a^^,^^b^	0.87 ± 0.11^b^	1.66 ± 0.46^c^^,^^d^	0.95 ± 0.15^d^
M30 (U/L)	21.72 ± 3.20^a^^,^^c^	47.55 ± 6.16^a^^b^	23.91 ± 5.86^b^	47.84 ± 5.44^c^^,^^d^	25.28 ± 3.45^d^
M65 (ng/L)	270.81 ± 16.22^a^^,^^c^	325.63 ± 24.49^a^^,^^b^	288.40 ± 19.88^b^	327.67 ± 16.50^c^^d^	289.96 ± 13.85^d^

Data are expressed as mean ± SD. Superscript letters indicate statistically significant differences between groups (*P* < 0.05, Tukey or Mann–Whitney U as appropriate):

^a^G1 vs G2,

^b^G2 vs G3,

^c^G1 vs G4,

^d^G4 vs G5,

^e^G3 vs G5 (no significant difference, thus not shown).

Specifically, the mean TOS levels increased from 12.05 ± 3.91 μmol H₂O₂ Eq./L in G1 to 16.52 ± 2.93 in G2 (*P* < 0.05) and 16.78 ± 2.18 in G4 (*P* < 0.05). Concurrently, TAS levels decreased significantly from 1.37 ± 0.20 mmol Trolox Eq./L in G1 to 1.10 ± 0.05 in G2 (*P* < 0.05) and 1.05 ± 0.19 in G4 (*P* < 0.05), reflecting impaired antioxidant capacity.

The OSI index, indicating oxidative balance, increased markedly in both irradiated groups (G2: 1.51 ± 0.31 AU, G4: 1.66 ± 0.46 AU) compared to G1 (0.90 ± 0.33 AU) (*P* < 0.05) for both comparisons.

Furthermore, serum M30 levels, indicating early apoptosis, rose significantly from 21.72 ± 3.20 U/L in G1 to 47.55 ± 6.16 in G2 and 47.84 ± 5.44 in G4 (*P* < 0.05). Similarly, M65 levels increased from 270.81 ± 16.22 ng/L in G1 to 325.63 ± 24.49 in G2 and 327.67 ± 16.50 in G4 (*P* < 0.05), consistent with increased cell death following irradiation.

Importantly, MEL supplementation in G3 and G5 groups reversed most of these alterations. For example, TOS levels decreased to 11.87 ± 0.89 in G3 and 12.25 ± 1.33 in G5 (*P* < 0.05 vs. G2 and G4, respectively), while TAS levels returned toward control values (G3: 1.38 ± 0.13, G5: 1.30 ± 0.12). The OSI index was similarly restored (G3: 0.87 ± 0.11, G5: 0.95 ± 0.15), with statistical significance (*P* < 0.05 vs. G2 and G4).

Additionally, M30 and M65 levels were significantly lower in G3 and G5 compared to G2 and G4, respectively (*P* < 0.05), suggesting MEL’s role in mitigating radiation-induced apoptosis. There was no significant difference in any biochemical parameter between G3 and G5 (*P* > 0.05), indicating comparable protective efficacy of MEL under both irradiation regimens.

According to the correlation analysis, serum M30 levels showed a strong correlation with cortical neuronal degeneration scores (Spearman ρ = 0.82, *P* < 0.001), while M65 correlated with cortical degeneration similarly (ρ = 0.82, *P* < 0.001). TOS and OSI exhibited moderate-to-strong correlations with overall histopathological severity (ρ = 0.68, *P* < 0.001). Full correlation coefficients are provided in Supplementary Table S1.

### Histopathologic findings

Comparative histological evaluation of radiation-induced cerebellum and cerebral cortex damage in all experimental groups is summarized in Table 3 and Table 4. Histopathological comparison between G2 and G4 radiotherapy groups and the G1 showed a statistically significant increase in tissue damage scores in both cerebral cortex and cerebellum (*P* < 0.05). The G3 and G5 groups showed a statistically significant reduction in histopathologic damage in both the cerebral cortex and cerebellum (*P* < 0.05) compared to the G2 and G4 groups treated with radiotherapy, respectively.

**Table 3:** Cerebellum histopathological scoring (median [min–max])

Parameter	G1	G2	G3	G4	G5
Neuronal degeneration	0 (0–1)^a^^,^^c^	2 (2–3)^a^^,^^b^	1 (1–2)^b^	2 (2–3)^c^^,^^d^	1 (1–2)^d^
Vascular dilatation	0 (0–1)^a^^,^^c^	2 (2–3)^a^^,^^b^	1 (1–2)^b^	2 (2–3)^c^^,^^d^	1 (1–2)^d^
Congestion	0 (0–1)^a^^,^^c^	2 (2–3)^a^^,^^b^	1 (1–2)^b^	2 (2–3)^c^^,^^d^	1 (1–2)^d^
Neuronal cell necrosis	0 (0–1)^a^^,^^c^	3 (2–3)^a^^,^^b^	1 (1–2)^b^	3 (2–3)^c^^,^^d^	2 (2–3)^d^

Data are presented as median (min–max). Superscript letters indicate statistically significant differences between groups (Mann–Whitney U test, *P* < 0.05):

^a^G1 vs G2,

^b^G2 vs G3,

^c^G1 vs G4,

^d^G4 vs G5,

^e^G3 vs G5 (no significant difference, thus not shown).

**Table 4:** Cerebral cortex histopathological scoring (median [min–max])

Parameter	G1	G2	G3	G4	G5
Neuronal degeneration	0 (0–1)^a^^,^^c^	2 (2–3)^a^^,^^b^	1 (1–2)^b^	2 (2–3)^c^^,^^d^	1 (1–2)^d^
Axonopathy	0 (0–1)^a^^,^^c^	2 (2–3)^a^^,^^b^	2 (1–3)^b^	2 (2–3)^c^^,^^d^	2 (1–2)^d^
Inflammation	0 (0–1)^a^^,^^c^	3 (2–3)^a^^,^^b^	2 (1–3)^b^	3 (2–3)^c^^,^^d^	2 (1–3)^d^
Dysplasia	0 (0–1)^a^^,^^c^	2 (2–3)^a^^,^^b^	2 (1–2)^b^	2 (2–3)^c^^,^^d^	2 (1–2)^d^
Vascular dilatation	0 (0–1)^a^^,^^c^	2 (2–3)^a^^,^^b^	1 (1–2)^b^	2 (2–3)^c^^,^^d^	1 (1–2)^d^
Congestion	0 (0–0)^a^^,^^c^	3 (2–3)^a^^,^^b^	1 (1–2)^b^	3 (2–3)^c^^,^^d^	2 (1–2)^d^

Data are presented as median (min–max). Superscript letters indicate statistically significant differences between groups (Mann–Whitney U test, *P* < 0.05):

^a^G1 vs G2,

^b^G2 vs G3,

^c^G1 vs G4,

^d^G4 vs G5,

^e^G3 vs G5 (no significant difference, thus not shown).

Moreover, no statistically significant difference in histopathological parameters was observed between G2 and G4 groups (*P* > 0.05), indicating comparable tissue effects between the two irradiation methods in the absence of MEL treatment (G2, G3).

**Fig. 1:**
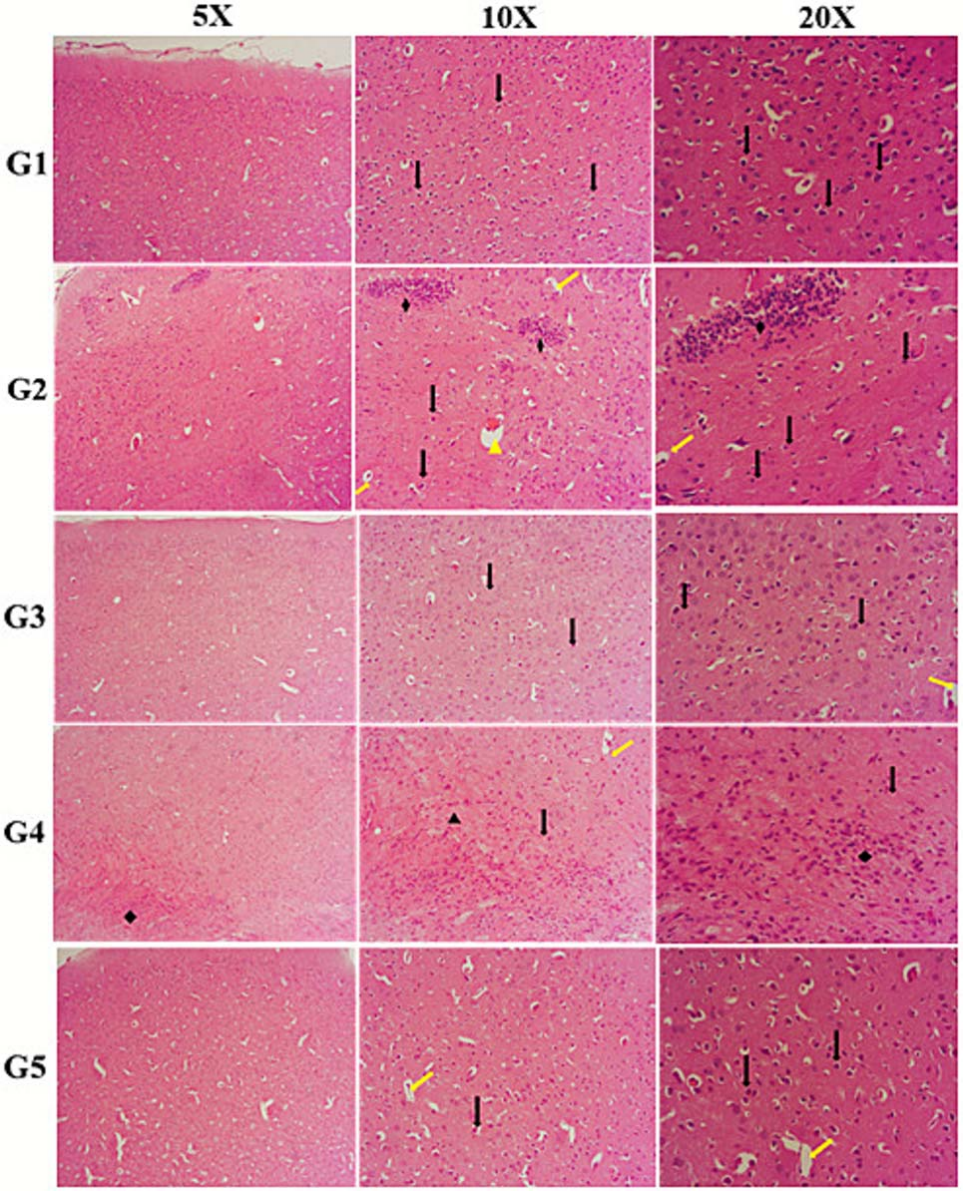
Representative histopathological images of cerebral cortex tissues from all experimental groups (G1–G5) at 5×, 10×, and 20× magnifications. G1 (control) shows normal cortical architecture with intact neuronal cells. G2 (FF irradiation) and G4 (FFF irradiation) exhibit marked pathological changes, including neuronal degeneration, pyknosis, axonopathy, vascular dilatation, congestion, and increased necrotic cell profiles. MEL-treated groups (G3: FF + MEL, G5: FFF + MEL) display milder histopathological alterations, with predominantly preserved neuronal morphology and fewer degenerative features, indicating a radioprotective effect of melatonin. Arrows indicate areas of degeneration, vascular changes, or neuronal damage.

Histologic examination of cerebral cortex sections revealed clear differences between the experimental groups. In the G1 (control) group, no histopathologic alterations were observed, and the cortical neuronal structures appeared normal. In contrast, G2 and G4 groups exhibited marked inflammatory changes, neuronal degeneration, axonopathy, vascular dilatation and congestion. In the G3 and G5 melatonin-treated groups, the cortical histoarchitectural appearance was largely preserved, with predominantly normal neuronal cells and only limited degenerative changes, indicating a protective effect of MEL. Representative light-microscopy images of the cerebral cortex for all groups are shown in Fig. 1.

**Fig. 2:**
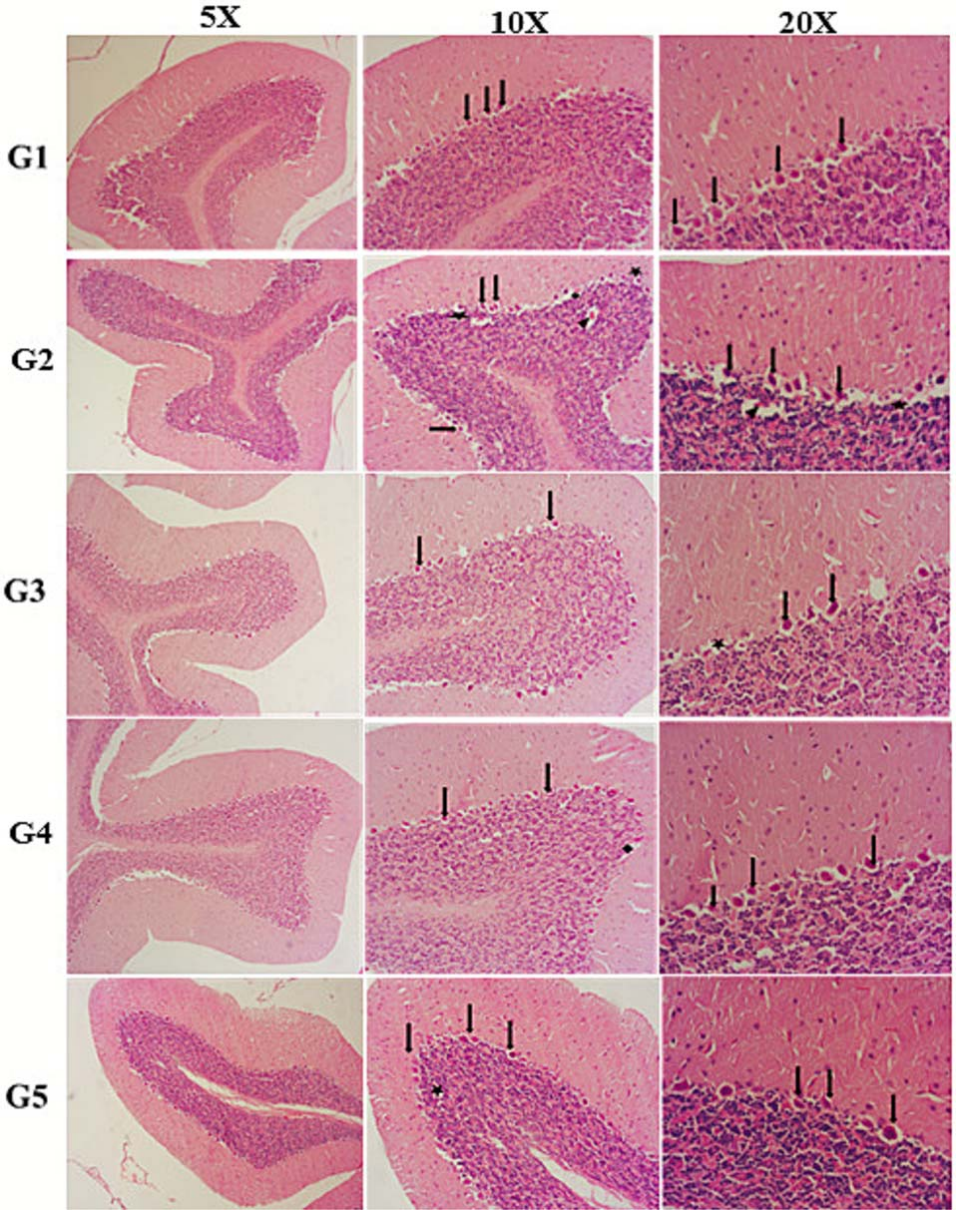
Representative histopathological images of cerebellar tissues from all experimental groups (G1–G5) at 5×, 10×, and 20× magnifications. G1 (control) shows normal cerebellar architecture with intact Purkinje cells and well-organized cortical layers. G2 (FF irradiation) and G4 (FFF irradiation) demonstrate prominent pathological alterations, including Purkinje cell degeneration, neuronal edema, vascular dilatation, congestion, and increased necrotic cell presence.MEL-treated groups (G3: FF + MEL, G5: FFF + MEL) exhibit markedly reduced histopathological damage, with preserved Purkinje cell integrity and fewer degenerative changes, indicating the radioprotective effect of melatonin. Arrows highlight regions of neuronal degeneration and vascular alterations.

Histologic analysis of cerebellum sections from the experimental groups revealed the following findings. No histopathologic changes were observed in the G1 group; Purkinje cells appeared normal. Representative light microscopy images of the G1 group are presented in Fig. 2. In the G2 and G4 groups, neuronal cell necrosis, neuronal degenerated purkinje cells, neuronal edema, vascular dilation, congestion were detected in cerebellum. The morphological form of the histological sections in the G3 and G5 groups resembled that of the G1 group, and the histopathological results were less severe than those observed in the G2 and G4 groups. G3 group compared with G2 group and G5 group compared with G4 group showed normal intact purkinje cells, mild neuronal edema, few degenerated neuronal cells. Histopathological findings of G3 and G5 groups are shown in Fig. 2. The compared images of cerebellum tissues getting from light microscopy for each groups showed in Fig. 2.

Histopathological evaluation of the cerebellum demonstrated significant differences among the experimental groups (Table 3). In G2 and G4 groups, neuronal degeneration, vascular dilatation, congestion and neuronal cell necrosis were markedly increased compared to the control group (G1) (*P* < 0.05). Conversely, MEL-treated groups (G3 and G5) showed significantly reduced scores for these parameters compared to their respective irradiation-only groups (G2 and G4) (*P* < 0.05). No significant differences were observed between G2 and G4, or between G3 and G5 (*P* > 0.05), indicating comparable effects of FF and FFF beams, while MEL co-administration (G3, G5) consistently reduced cerebellar damage.

Histopathological evaluation of the cerebral cortex revealed significant changes among the experimental groups across all assessed parameters. As illustrated in Table 4, the severity of neuronal degeneration, vascular dilatation, congestion and neuronal cell necrosis varied markedly between groups, with FF and FFF radiation groups (G2 and G4) exhibiting a higher prevalence of moderate-to-severe damage compared to the control group (G1). Notably, MEL co-administration (G3 and G5) appeared to attenuate these effects, particularly in reducing severe degeneration and necrosis.

A comparative summary of six histopathological markers—neuronal degeneration, axonopathy, inflammation, dysplasia, vascular dilatation and congestion—is provided in Table 4. Consistently across parameters, the control group predominantly exhibited ‘none’ or ‘minimal’ scores, while irradiated groups (G2–G5) showed a rightward shift toward higher severity grades. Among these, MEL-treated groups (G3 and G5) demonstrated a relative decrease in the frequency of severe histopathological changes. Overall group differences were statistically significant (Kruskal–Wallis, *P* < 0.001), and pairwise comparisons are detailed in Table 4.

## DISCUSSION

Although radiotherapy is a major treatment modality for central nervous system malignancies, it is also associated with various adverse effects on healthy brain tissue [26]. Despite its widespread clinical use, the mechanisms underlying radiation-induced brain injury at varying doses and dose rates, especially in the early post-irradiation phase, are still poorly understood. Therefore, the assessment of radiation-induced damage to brain parenchyma and cerebral vascular system as a function of dose and dose rate is of significant radiobiological importance. In this study, we specifically focused on early-phase responses (48 hours post-irradiation), which allows for evaluation of acute oxidative and apoptotic changes, but does not capture long-term remodeling or late radiation effects. Also, experimental brain injury models were established using FF and FFF beams applied to the cerebral cortex and cerebellum of rats. Both biochemical and histopathological analyses were conducted to assess the extent of radiation-induced injury in brain tissues and serum samples.

Previous experimental and literature-based evidence suggests that ionizing radiation induces the formation of free radicals that disrupt the balance between prooxidant and antioxidant defense systems. This disruption leads to oxidative stress, causing significant cellular damage to DNA, proteins and membrane lipids [27]. ROS contribute to oxidative stress by altering intracellular antioxidant enzyme levels and reducing overall antioxidant capacity. During radiotherapy targeting the central nervous system, increased ROS production in neuronal tissue exacerbates oxidative damage. Neuronal membranes, which exhibit high oxygen consumption and lipid content, are particularly susceptible to free radical-induced damage. As a result, increased lipid peroxidation is frequently observed in irradiated brain tissue [28].

In this study, serum samples were analyzed to assess the levels of M30 (apoptosis marker), M65 (necrosis marker), TAS, TOS and OSI following irradiation with FF and FFF beams. Biochemical data showed that ionizing radiation significantly elevated M30 and M65 levels in both FF and FFF groups compared to the control group. However, MEL administration before radiotherapy in the FF + MEL and FFF + MEL groups led to a statistically significant reduction in M30 and M65 levels compared to their counterparts exposed to radiation alone (FF and FFF groups). These findings suggest that MEL exerts a significant radioprotective effect by attenuating radiation-induced apoptosis and necrosis, as reflected by suppressed M30 and M65 levels. Furthermore, no statistically significant difference in M30 and M65 levels was observed between FF and FFF groups, suggesting comparable effects on early radiation-induced cell death markers regardless of dose rate.

Our radiobiological results from serum analysis are in agreement with previous *in vitro* studies reporting similar cellular responses following FF and FFF irradiation in various cancer cell lines [17, 29, 30] However, some *in vitro* studies have shown that increasing instantaneous dose rate does not significantly affect cell survival [31, 32]. These discrepancies in the literature can be attributed to differences in experimental design, cell type and inherent radiosensitivity [33].

Analysis of TOS, TAS and OSI levels in serum samples was performed to assess the oxidative stress response across different dose rates and radiotherapy modalities. While no statistically significant difference was observed between FF and FFF groups, both treatment groups showed significant changes compared to the control group. Notably, radiotherapy caused an increase in TOS levels and a decrease in TAS levels, indicating increased oxidative stress.

Importantly, administration of MEL before irradiation led to significant improvements in these parameters. The reduction in TOS and restoration of TAS levels suggest that MEL effectively inhibits oxidative damage caused by ionizing radiation. These results support the hypothesis that free radicals generated during radiotherapy play a central role in disrupting the oxidant-antioxidant balance.

Consistent with our findings, Benoit *et al.* showed that MEL significantly increased antioxidant defenses after irradiation [34]. Correlation analysis further demonstrated that serum M30 and M65 levels strongly reflected cortical neuronal degeneration, supporting their potential as surrogate biomarkers of radiation-induced brain damage. Furthermore, moderate-to-strong associations of TOS and OSI with histopathological severity reinforce the central role of oxidative stress in mediating tissue injury. Similarly, the TAS and TOS results reported in our study are consistent with previous literature [35] MEL has been shown to facilitate tissue repair, protect healthy tissue and reduce oxidative stress by neutralizing free radicals. Our results also confirm that MEL has a radioprotective effect against both FF and FFF irradiation attributed to its potent antioxidant properties [15, 35, 36].

Macroscopic and histopathological analyses are the most reliable methods for assessing damage to brain tissue exposed to radiation. In this study, various early radiation-related damage on cerebral cortex and cerebellum, caused by FF and FFF beams, were evaluated using histopathological parameters. Interestingly, cerebellar damage scores tended to be more pronounced than cortical alterations, particularly in Purkinje cells, consistent with previous evidence indicating the higher radiosensitivity of these neuronal populations. [37] Histological sections of cerebellum tissue revealed neuronal degeneration, axonopathy, inflammation, dysplasia, vascular dilatation and congestion. In cerebral cortex tissues, findings of neuronal degeneration, vascular dilatation, congestion and neuronal cell necrosis were significantly observed in both FF and FFF groups. These histopathological findings showed a significant improvement with MEL application in the FF + MEL and FFF + MEL groups. The results of this study show that MEL administered concurrently with radiotherapy significantly reduces oxidative stress and exhibits a neuroprotective effect, thus enhancing histopathological recovery in early rat brain injuries.

Based on the biochemical and histopathological findings obtained in this study, it was demonstrated that radiotherapy-induced oxidative stress contributes significantly to nervous tissue damage. MEL treatment exhibited both anti-inflammatory and antioxidant properties and effectively reduced early-stage ionizing radiation-induced oxidative stress in brain tissues and serum samples. While the neuroprotective effects of various antioxidants, including MEL, are well documented, the radiobiological effects of high instantaneous dose rates, such as FFF beams, on brain tissue remain unclear [38]. Despite extensive *in vitro* studies investigating FFF-induced radiobiological effects, *in vivo* evidence remains limited. For example, Lohse *et al.* reported that FFF irradiation significantly reduced cell survival in certain experimental models [39]. In contrast, other *in vitro* studies found no significant difference in survival when high instantaneous dose rate irradiation was applied [29]. These inconsistencies underscore the need for further research to clarify the radiobiological effects of FFF irradiation on tissue viability. Overall, the current literature lacks sufficient experimental and clinical data addressing the biological impact of FFF irradiation, especially in the context of brain tissue response.

Given the increasing clinical use of FFF beams, especially in stereotactic radiosurgery (SRS) and stereotactic body radiotherapy (SBRT), it is crucial to determine whether FF and FFF beams differ in their biological effects. To our knowledge, this is among the first *in vivo* studies directly comparing FF and FFF beams in healthy rat brain tissue, with a parallel evaluation of MEL’s radioprotective role. In our study, FF and FFF irradiation produced comparable biochemical and histopathologic results in both brain tissue and serum samples. These findings are consistent with the majority of available *in vitro* studies, with the exception of the results reported by Lohse *et al.* [30]

A limitation of our study is that it focused on specific early-stage biochemical and histopathological markers to assess the neuroprotective effects of MEL against FF and FFF-induced brain injury. Additional limitations include the use of a single high-dose fraction, which differs from the fractionated regimens typically applied in clinical practice, and the exclusive use of female Wistar albino rats, which may limit generalizability. Future research is needed to evaluate the long-term effects of MEL in *in vivo* models of radiation-induced brain injury at different dose levels and time points.

## CONCLUSION

This study demonstrated that radiotherapy induces significant neural tissue damage by enhancing oxidative stress in the early post-irradiation period. MEL administration provided potent radioprotective effects through reducing oxidative stress and improving histopathological findings in rat brain tissues. Moreover, both FF and FFF beams showed a consistent impact biochemically and histopathologically on the tissues of the cerebral cortex and cerebellum.

Importantly, no significant difference in early biochemical and histopathological damage was observed between FF and FFF beams, suggesting that both modalities exert comparable short-term effects on brain tissue under the conditions studied. Overall, the results of this study contribute to the limited *in vivo* data on the biological effects of FFF irradiation and emphasize the potential of MEL as a radioprotective agent. Future studies should investigate long-term outcomes under fractionated regimens and explore whether the radioprotective efficacy of MEL observed in this preclinical model can be translated into clinical practice.

## Supplementary Material

Supplementary_Table_S1_rraf078

## CONFERENCE PRESENTATION

This manuscript has not been presented at any conference.
