# Supplementary material for: Early radiobiological effects of flattening filter (FF) and flattening filter-free (FFF) beams and the radioprotective role of melatonin in rat brain: a preclinical study
Source: J Radiat Res. 2025 Dec 23;67(1):59–67. doi: 10.1093/jrr/rraf078 (PMC12856033; doi:10.1093/jrr/rraf078)
Supplement: Supplementary_Table_S1_rraf078 [file supplementary_table_s1_rraf078.docx]

# Supplementary Table S1

Spearman correlation coefficients (ρ) between serum biomarkers (TAS, TOS, OSI, M30, M65) and histopathological parameters (cerebral cortex and cerebellum). Statistically significant associations were observed, with M30 and M65 strongly correlating with neuronal degeneration, TOS/OSI showing moderate-to-strong positive correlations with overall histopathological severity, and TAS showing weak-to-moderate inverse correlations with damage parameters. p<0.05 was considered statistically significant.

| **Serum Biomarker** | **Cortical Neuronal Degeneration (ρ, p)** | **Cerebellar Degeneration (ρ, p)** | **Overall Histopathological Severity (ρ, p)** |
| --- | --- | --- | --- |
| TAS | -0.42, 0.02 | -0.38, 0.04 | -0.40, 0.03 |
| TOS | 0.65, <0.001 | 0.62, <0.001 | 0.68, <0.001 |
| OSI | 0.67, <0.001 | 0.64, <0.001 | 0.68, <0.001 |
| M30 | 0.82, <0.001 | 0.78, <0.001 | 0.81, <0.001 |
| M65 | 0.82, <0.001 | 0.79, <0.001 | 0.82, <0.001 |
